# Freezing during tapping tasks in patients with advanced Parkinson’s disease and freezing of gait

**DOI:** 10.1371/journal.pone.0181973

**Published:** 2017-09-08

**Authors:** Arnaud Delval, Luc Defebvre, Céline Tard

**Affiliations:** 1 Univ. Lille, INSERM U1171 Degenerative & Vascular Cognitive Disorders, Lille, France; 2 Clinical Neurophysiology Department, Lille University Medical Center, Lille, France; 3 Lille Center of Excellence for Neurodegenerative Diseases, LiCEND, Univ Lille, Lille, France; 4 Neurology and Movement Disorders Department, Lille University Medical Center, Lille, France; Oslo Universitetssykehus, NORWAY

## Abstract

**Introduction:**

Parkinson’s disease patients with freezing of gait also experience sudden motor blocks (freezing) during other repetitive motor tasks. We assessed the proportion of patients with advanced PD and freezing of gait who also displayed segmental “freezing” in tapping tasks.

**Methods:**

Fifteen Parkinson’s disease patients with freezing of gait were assessed. Freezing of gait was evaluated using a standardized gait trajectory with the usual triggers. Patients performed repetitive tapping movements (as described in the MDS-UPDRS task) with the hands or the feet in the presence or absence of a metronome set to 4 Hz. Movements were recorded with a video motion system. The primary endpoint was the occurrence of segmental freezing in these tapping tasks. The secondary endpoints were (i) the relationship between segmental episodic phenomena and FoG severity, and (ii) the reliability of the measurements.

**Results:**

For the upper limbs, freezing was observed more frequently with a metronome (21% of trials) than without a metronome (5%). For the lower limbs, the incidence of freezing was higher than for the upper limbs, and was again observed more frequently in the presence of an auditory cue (47%) than in its absence (14%).

**Conclusion:**

Although freezing of the lower limbs was easily assessed during an MDS-UPDRS task with a metronome, it was not correlated with the severity of freezing of gait (as evaluated during a standardized gait trajectory). Only this latter was a reliable measurement in patients with advanced Parkinson’s disease.

## Introduction

Freezing of gait (FoG) has been defined as the “absence or marked reduction of forward progression of the feet despite the intention to walk” [1]. FoG is frequently reported in Parkinson’s disease (PD) [2], and has been identified as an independent risk factor for falls [3, 4] and impaired quality of life [5]. The “gold standard” for FoG measurement is a clinical evaluation of video recordings of ambulating patients by one, two or three raters (who ideally should be experts in the assessment of FoG) [6]. One of the main problems in FoG measurement (as highlighted by Snijders et al. [7]) is that an examination in a lab environment or the physician’s office can temporarily suppress a patient’s FoG. This failure to elicit the FoG phenomenon poses a problem for physicians, who need to base their evaluation on observations in the consulting room; this is why researchers have looked for more easily detectable equivalents of FoG or festination in effectors other than the lower limbs.

Finger tapping may be an equivalent of FoG on upper limbs. Indeed, finger tapping provokes “manual motor blocks” which are correlated with patients’ gait impairments. These motor blocks also called segmental “freezing episodes” may occur in repetitive upper and lower limb movements [8]. These blocks are commonly assessed by using items 3.4 and 3.8 of the Movement Disorder Society-sponsored revision of the Unified Parkinson's Disease Rating Scale (MDS-UPDRS) [9]). It has been reported that these episodes of upper limb freezing are correlated with FoG scores, independently of disease severity or cognitive impairment [10–12]. Since the initial reports, many studies have used finger-tapping tasks to assess equivalents of FoG in the upper limbs [13–17]. Equivalents of FoG in the lower limbs are currently being used to study the physiopathology of FoG in neuroimaging experiments (e.g. functional MRI) because studying gait directly is not possible inside the bore of an MRI machine [18]. Numerous studies having mainly evaluated the occurrence of segmental (hand or foot) freezing during various protocols (for a review, see [19]), including either unilateral hand or finger tapping without cues, with cues [13, 20–22], during bilateral hand or finger tapping[13–16, 23–25]. Studies on lower limbs also used various protocols including mainly bilateral tapping or pedaling, associated with cues [15] or without [26–28] or with virtual reality protocols [23, 25–27].

The use of external cue can be very useful to trigger freezing [23, 26, 29, 30]. We previously showed that the use of an external cue at an appropriate frequency was associated with an elevated occurrence of episodic rhythmic disorders (at around 4 Hz) [30]. However, those segmental freezing phenomena have not been studied in patients with advanced PD who suffer from residual off-FoG, despite optimized dopatherapy. We therefore sought to determine whether these freezing phenomena were also relevant in this particular patient population.

The primary objective of the present study of advanced PD patients presenting FoG was to determine the occurrence of episodic segmental freezing phenomena on upper and lower limbs during commonly used tests (items 3.4 and 3.8 of the MDS-UPDRS [9]) in the presence and absence of a metronome set to 4 Hz. The secondary objective was to test the reliability of these phenomena between 2 sessions and their relationship with the severity of FoG.

We hypothesized that freezing equivalents would be more frequent in the presence of a metronome since a metronome could better trigger these segmental freezing episodes [30]. Indeed, freezing episodes during diadochokinetic tasks mainly occur at between 3 and 5 Hz [30]. As a secondary objective, we expected that the reliability of these measurements between 2 sessions would be high. Given that equivalents of freezing have been observed in advanced PD patients in previous literature (see [19]), we also expected that freezing equivalent will be present in all patients presenting actual FoG and highly correlated with its severity as suggested by several authors [10, 29, 31].

## Material and methods

### Participants

Patients with PD (diagnosed according to UK PDS Brain Bank criteria [32]) were enrolled from the active case file of the Movement Disorders Department at Lille University Medical Center (Lille, France) (Table 1). The patients were selected on the basis of their answer to item 3 of the FoG questionnaire [33]. To check that FoG was indeed not fully controlled by medication, we asked patients to perform 540° turns to the left and to the right (at normal and maximum speeds) when they were in the “on” state (i.e. while taking their usual medications). Only patients with clinically confirmed, treatment-refractory “off” FoG (despite having a stable medication regimen for at least the previous 3 months) were eligible for inclusion.

**Table 1:** Clinical characteristics of the study population.

Subject	Age (years)	MOCA (/30)	Gender	BMI (kg/m2)	Disease duration (years)	FOG questionnaire	item 2.13 MDS-UPDRS	item 3.11 MDS-UPDRS	MDS-UPDRS-3	Hoeh and Yahr 'on drug'
1	57	28	1	27	18	11	2	0	38	2.5
2	72	22	1	22	15	11	2	2	38	2
3	71	27	2	30	7	12	3	2	46	3
4	69	19	1	22	15	13	1	1	35	2
5	72	21	1	22	9	14	3	2	40	2
6	59	23	1	31	10	16	1	2	42	2
7	77	25	2	22	4	11	2	2	55	3
8	65	27	1	25	15	18	0	0	24	2
9	73	27	1	31	4	12	2	2	42	3
10	71	21	2	26	17	15	1	0	46	2
11	53	29	1	26	15	17	1	0	58	2
12	74	29	1	23	14	15	3	2	41	3
13	66	25	2	22	13	18	3	3	57	3.5
14	60	20	1	27	12	11	1	1	39	3
15	58	24	1	24	4	18	3	0	42	4
Median	69	25		25	13	14	2	2	42	2.5
Quartile 1	60	22		22	8	12	1	0	39	2
Quartile 3	72	27		27	15	17	3	2	46	3

Cognitive status was evaluated using the Montreal Cognitive Assessment [35] and freezing was evaluated with items 2.13 and 3.11 of the MDS-UPDRS [9] and with the FoG questionnaire [33].

The exclusion criteria included the inability to walk alone, the use of deep brain stimulation, the presence of neurological disorders other than PD, and ongoing major depression.

The study was approved by the local institutional review board (CPP Nord-Ouest IV, Lille, France; reference 13/41, n°2013-A00737-38) and promoted by Lille University Medical Center. Signed informed consent was obtained from all patients in accordance with the institutional ethics committee board.

### Experimental design

The study comprised a session at baseline and a second session one week later. Two conditions (with and without a metronome) were applied in each session, in random order. All evaluations were made between 9.00 am and 10.30 am in the “on-drug” condition, between 90 and 120 minutes after the usual dopaminergic intake. Indeed, most of them would have been unable to perform the tests in “off-drug” condition and still presented FoG in “on-drug” condition (see inclusion criteria).

### Clinical evaluations: FoG evaluation

We measured the duration of FoG (and then calculated the percentage time with FoG) during a standardized FoG trajectory comprising gait initiation, turning (360- and 540-degree turns at the preferred speed and at maximum speed), walking through a narrow passage, and dual-tasking (walking while counting backwards in threes) [34]. The trajectories were filmed with a video camera. Offline, two raters (AD and CT) determined the completion time and the time with FoG for each part of the trajectory.

### Movement analysis

Kinematic parameters were recorded using a VICON 3D motion analysis system with eight infrared cameras (sampled at 100 Hz). Reflective spheres were placed on the third phalanx of the index for the hand task, and on the calcaneus for the foot task.

### Upper and lower limbs tasks (S1 Video)

The hand task: Each hand was tested separately. We demonstrated the task, but did not continue to perform the task while the patient was tested. We instructed the patient to tap the index finger on the thumb (i) as big and as fast as possible (ii) as big as possible in time with an auditory cue (a metronome set to 4 Hz). The tap (index finger on the thumb) had to be in time with the metronome sound. The order of the two conditions was pseudo-random. At least 30 productions on each body side were recorded.

The foot task: The patient sat in a straight-back chair, both feet on the floor. We demonstrated the task, but do not continue to perform the task while the patient is tested. We then instructed the patient to place the foot on the ground and then raise and stomp the foot on the ground (i) as high and as fast as possible, (ii) to stomp the foot on the ground as high as possible in time with the metronome set to 4 Hz. Again, the order of the two conditions was pseudo-random, and at least 30 productions on each body side were recorded.

### Data processing

A series of 30 productions were selected for each effector and each condition. For each production, the peak-to-peak interval was determined semi-automatically using a home-made matlab script and corrected manually by the examiner if obviously erroneous (peaks and nadir on sagittal plane were then picked manually). This process yielded the instantaneous frequency and amplitude of each tap.

### Definition of episodic events (Fig 1)

Freezing episodes of the hands and feet were defined as involuntary arrests of repetitive movement, i.e. an amplitude less than half the mean amplitude in the test for at least 0.5 seconds (as defined in the literature [11, 17]).

**Fig 1:**
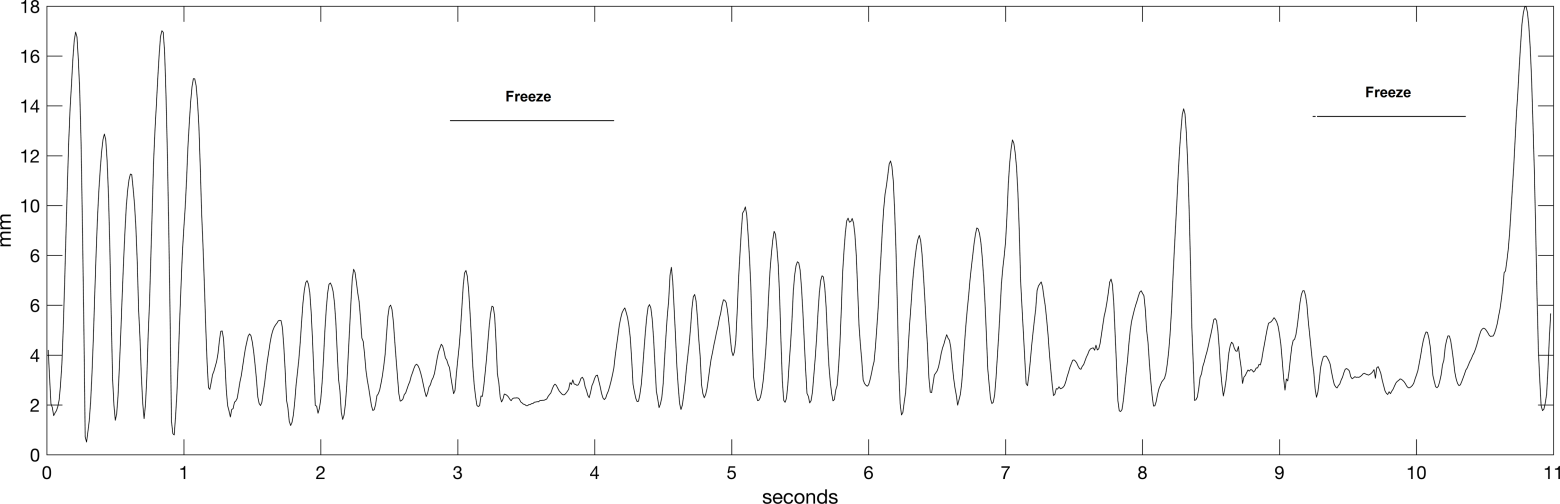
A patient presented two episodes of freezing while performing the lower-limb tapping task in the presence of auditory cueing. The position of the right heel marker (in the sagittal plane) is shown here.

Voluntary stops were excluded by careful examination of the video-taped trials (raters: CT and AD). Depending on the number of episodes of freezing, each trial was further classified as a “freezing trial” or a “no-freezing trial”.

### Definition of rhythmicity parameters

The frequency and amplitude of tapping, and the coefficients of variation (CVs) for the amplitude and duration of the tapping (defined as the standard deviation (SD) of the 30 instantaneous amplitudes divided by the mean of the 30 instantaneous amplitudes) were computed.

### Statistical analysis

Statistical analysis was performed using SPSS software (version 16.0, IBM Corp., Armonk, NY). The threshold for statistical significance was set to p<0.05.

The primary study criterion was the frequency of episodic events for each condition (the presence or absence of auditory cueing) and each effector (upper limb or lower limb). Categorical data were compared using chi-squared tests. Model assumptions (normality of residuals and homoscedasticity) were checked, and data were rank-transformed if the assumptions were not satisfied. To test our primary objective (occurrence of freezing of effectors in PD freezers and elicitation by auditory cueing), we used a general linear model (analysis of variance with repeated measures) with a between-subject factor (the presence or absence of auditory cueing) and a within-subject factor (the effector). If the interaction was significant, post-hoc comparisons were performed using the Bonferroni correction.

The secondary criteria included the reliability of the measurements, and the relationship between episodic events and severity of FoG. For reliability, Cronbach’s alpha was computed. Either Pearson’s or Spearman’s correlation coefficient was calculated as a guide to the relationship between the duration of freezing episodes (in all trials performed) and the corresponding clinical data on FoG.

## Results

### Occurrence of FoG

Fifteen patients were included in the study. The patients’ demographic and clinical characteristics are summarized in Table 1. The patients had similar levels of motor performance in the two sessions. All 15 patients presented with FoG. The median (interquartile range IQR) time with FoG during the FoG trajectory was 23 (6–51) seconds. The median ratio (IQR) between the time with FoG and the trajectory completion time was 0.35 (0.08–0.56).

### Occurrence of freezing episodes for the two effectors

In the hand task, 3 of the 15 patients displayed episodes of freezing in the absence of an auditory cue and 7 displayed episodes in the presence an auditory cue. For the foot task, 9 of the 15 patients displayed episodes of freezing in the absence of an auditory cue and 11 displayed episodes in the presence an auditory cue. We observed an effect of the effector (p = 0.02 in a chi- squared test) but not an effect of cueing (p = 0.2 in a chi- squared test).

For the hand, freezing occurred in 5% of the trials without auditory cueing and in 21% of the trials with auditory cueing. For the foot, freezing occurred in 14% of the trials without auditory cueing and in 47% of the trials with auditory cueing. We observed an effect of auditory cueing (p<0.001) and an effect of the effector (p<0.001).

The mean ± SD duration of episodes of freezing (with pooled data for the two effectors because there was no difference between the hand and the foot) was 0.84 ± 0.45 s without auditory cueing and 1.10 ± 0.60 s with auditory cueing; this difference was not significant).

### Rhythmicity parameters (Table 2)

For both effectors, the tapping frequency was significantly higher in the presence of auditory cueing (p<0.001). Regardless of the condition, the tapping frequency was lower for the lower limbs than for the upper limbs (p<0.001). The amplitude was lower in the presence of auditory cueing (p<0.001). There was no effect of the effector on amplitude, although a significant cueing x effector interaction was observed (p = 0.001): for the foot task, the amplitude was significantly lower in the presence of auditory cueing. The variability of the amplitude and the duration was higher with auditory cueing (p<0.001), and the effects were more marked for the feet (p<0.001 for the cueing x effector interaction, for both amplitude and duration).

**Table 2:** Rhythmicity parameters.

	hands	hands metronome	feet	feet metronome	effector	metronome	Interaction
Frequency (mean/SD) Hz	4.0/0.9	4.5/0.9	3.1/0.7	3.8/0.7	p<0.001	p<0.001	0.428
Amplitude mm	25 /13	21/12	29/21	16/14	p<0.001	0.748	p = 0.001
CV duration	0.11/0.09	0.10/0.08	0.07/0.05	0.15/0.12	0.688	p<0.001	p<0.001
CV amplitude	0.19/0.08	0.21/0.12	0.22/0.12	0.34/0.16	p<0.001	p<0.001	p<0.001

### Reliability parameters

When comparing the two sessions, Cronbach’s alpha was 0.75 for the ratio between the time with FoG and the FoG trajectory completion time, 0.72 for time with FoG, and 0.36 for the percentage of trials with FoG.

### Correlations between episodic events, impaired rhythm generation, and the severity of FoG

Whatever the effector, neither the mean time spent with freezing during segmental tasks with auditory cueing nor the proportion of trials with FoG was correlated with the time with FoG during FoG trajectory, ratio between the time with FoG and the FoG trajectory completion time or other clinical variables including FoG questionnaire (see Table 1). Time spent with FoG was positively correlated with the MDS-UPDRS item 2.13 and 3.11 scores (Rho = 0.54, p<0.01; Rho = 0.63, p<0.001, respectively) but not with FoG questionnaire.

## Discussion

The two main findings of the present study are that commonly used tests (items 3.4 and 3.8 of the MDS-UPDRS[9]) can trigger freezing—mainly in the lower limbs. The use of a metronome increased the occurrence of motor blocks by a factor of two or three, although the number of patients who did not present freezing at all during the item 3.8 test was not significantly lower in the presence of auditory cueing. Furthermore, we evaluated the intersession reliability of the “gold standard” clinical test (the FoG trajectory) [36] and the occurrence of freezing during segmental tasks. The intersession reliability was low (Cronbach’s alpha <0.8 in all cases), making these tasks difficult to use as primary criteria in interventional studies. However, the ratio between the time with FoG and the FoG trajectory completion time seems to be the most reliable parameter—in accordance with a literature report [37].

### Use of auditory cue to trigger freezing

It has already been reported that the use of a metronome can trigger freezing. In 2006, Moreau et al. [29] showed that patients suffered from FoG more frequently when a metronome was set at 140% of their normal pace. The application of a metronome can be considered as a stressful dual-task condition [38]. One can hypothesize that “mental collapse” leads to freezing; indeed, the stress induced by the metronome and the freezer patients’ difficulties in simultaneously controlling limb movement may trigger FoG. An alternative hypothesis is that the “dual task” increased freezing in our patients because of the latter’s limited ability to distribute attentional resources, due to lack of neural reserve (also called interference hypothesis of freezing [39]). In our study, we didn’t observe any correlation between freezing and the MoCA score that is a very global evaluation of cognition and not specific attentional components that could be more specific of freezing deficits [40]. Furthermore, the potential role of anxiety during the metronome condition should not be overlooked since it is highly prevalent in freezers [41] and can lead to freezing [42]. In the present study, the single imposed frequency (4 Hz, previously demonstrated to trigger freezing [30]) was higher than the patient’s self-generated frequency. Auditory cueing appears to increase the priority given to maintaining frequency because the amplitude decreased for the feet (but not for the hands). The prioritization of frequency might also explain the freezing phenomenon, given that the imposition of low-amplitude movements triggers FoG more easily [43, 44]. However, this was not confirmed in our upper limb task; during the trials without cueing, patients were instruction to perform the movement as big and as fast as possible. This condition was associated with great variability of the spatiotemporal parameters–especially when a higher frequency was imposed. The elevated variability may also have triggered freezing [45]. The instruction given to the patient is a crucial point. Asking to tap “as big/high as possible” can alleviate freezing since freezing is more frequent for low amplitude movements [44], although in the walking task, no instruction about step amplitude was given. This could explain -in part- differences between the 2 tasks.

### Occurrence of freezing according to the effectors

Lastly, we were very surprised to observe that hand and foot freezing phenomena occurred less frequently than FoG itself—even under stressed (cued) conditions. Our definition of freezing was based on a decrease in amplitude (i.e. severe hypokinesia) for at least 0.5 s; this might not be totally specific for freezing phenomena. In the absence of PD, freezing episodes of the upper or lower limbs are rarely observed in elderly patients [30]. However, this does not explain the differences observed when comparing upper limb and lower limb effectors. Some researchers have suggested that freezing is a somatotopic task because some patients freeze during activities such as speaking and writing [16]. This hypothesis is strengthened by the fact that all 15 patients presented FoG during an on-medication FoG trajectory but only 11 experienced freezing during a 4 Hz foot-tapping task. In early-stage PD, the production of bimanual movements in time with a metronome was a very sensitive freezing trigger [30]. However, this trigger might be inadequate in advanced PD patients, who present FoG and lower-limb freezing more frequently. It may be that different mechanisms and neural bases are involved. In summary, FoG occurs more frequently than either upper-limb or lower-limb freezing in advanced PD patients.

### Limitations

No control group (non-freezer patients) was recorded. Indeed, in studies involving rhythmic tasks of either upper limbs or lower limbs, there is large evidence that freezing is more frequent in patients presenting FoG compared with patients without FoG **[8,13,16,21,29–31]**.

We chose to quantify FoG using time spent with FoG during a standardized trajectory as our main quantification of FOG in our patients rather than other measurement such as FoG questionnaire. Indeed, all our patients presented by definition severe FoG with a high median score (14) with low interquartile interval at the FoG questionnaire (12–17). However, we didn’t find any additional correlation between FoG questionnaire and segmental freezing measurements.

We chose to test unilateral rhythmic movements (items 3.4 and 3.8 of the MDS-UPDRS) since they are used daily by clinicians to evaluate segmental akinesia, hypokinesia or bradykinesia. However, bimanual movement such as antiphase movements [46] or virtual reality [23,47] could have been more efficient to trigger freezing but were less commonly used in clinical evaluations.

## Conclusion

In patients with advanced PD and FoG, freezing of the upper and lower limbs can be observed in tapping tasks (especially in the presence of auditory cueing) but with a weak occurrence. The use of a metronome at 4 Hz can be useful to better trigger freezing even in a simple tapping task of the foot or the hand. However the occurrence of segmental freezing was less reliable than the FoG observed during a FoG trajectory and not correlated with FoG measurement. Then, the consideration of segmental freezing as tools or equivalents of FoG for studying freezing of gait should be used cautious since pathophysiology of these phenomena may differ.

## Supporting information

S1 Video A patient performing the upper and lower limb tasks. (MP4)
